# The effect of the apolipoprotein E ε4 allele and olfactory function on odor identification networks

**DOI:** 10.1002/brb3.3524

**Published:** 2024-05-03

**Authors:** Conner Frank, Abigail Albertazzi, Claire Murphy

**Affiliations:** ^1^ SDSU/UC San Diego Joint Doctoral Program in Clinical Psychology San Diego California USA; ^2^ Department of Psychology San Diego State University San Diego California USA; ^3^ Department of Psychiatry University of California San Diego La Jolla California USA

**Keywords:** Alzheimer's, APOE, functional connectivity, network connectivity, olfaction

## Abstract

**Introduction:**

The combination of apolipoprotein E ε4 (ApoE ε4) status, odor identification, and odor familiarity predicts conversion to mild cognitive impairment (MCI) and Alzheimer's disease (AD).

**Methods:**

To further understand olfactory disturbances and AD risk, ApoE ε4 carrier (mean age 76.38 ± 5.21) and ε4 non‐carrier (mean age 76.8 ± 3.35) adults were given odor familiarity and identification tests and performed an odor identification task during fMRI scanning. Five task‐related functional networks were detected using independent components analysis. Main and interaction effects of mean odor familiarity ratings, odor identification scores, and ε4 status on network activation and task‐modulation of network functional connectivity (FC) during correct and incorrect odor identification (hits and misses), controlling for age and sex, were explored using multiple linear regression.

**Results:**

Findings suggested that sensory‐olfactory network activation was positively associated with odor identification scores in ε4 carriers with intact odor familiarity. The FC of sensory‐olfactory, multisensory‐semantic integration, and occipitoparietal networks was altered in ε4 carriers with poorer odor familiarity and identification. In ε4 carriers with poorer familiarity, connectivity between superior frontal areas and the sensory‐olfactory network was negatively associated with odor identification scores.

**Conclusions:**

The results contribute to the clarification of the neurocognitive structure of odor identification processing and suggest that poorer odor familiarity and identification in ε4 carriers may signal multi‐network dysfunction. Odor familiarity and identification assessment in ε4 carriers may contribute to the predictive value of risk for MCI and AD due to the breakdown of sensory‐cognitive network integration. Additional research on olfactory processing in those at risk for AD is warranted.

## Graphical abstract

Olfactory function is impaired in Alzheimer's disease and apolipoprotein ε4 carriers at risk for Alzheimer's disease; however, underlying network alterations have been underexplored. This study suggests that impaired odor familiarity and odor identification ability in ε4 carriers predict disruption in the task‐modulated functional connectivity of sensory‐olfactory processing networks assessed with independent components analysis.

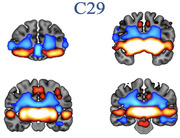

## 1 INTRODUCTION

Alzheimer's disease (AD) is a progressive neurodegenerative disease that currently affects 3.65 million older adults in America and is expected to affect 9.3 million older adults by 2060 (Brookmeyer et al., 2018). There is significant evidence that the AD disease process begins significantly earlier than cognitive deficits are detectable by gold‐standard and widely available neuropsychological assessments (Sperling et al., 2014, 2011; Vermunt et al., 2019), which presents significant challenges with developing effective treatments for AD due to the difficulty identifying those at risk of AD development prior to the onset of advanced neurodegeneration.

The most established genetic risk factor for AD is the apolipoprotein E ε4 (ApoE ε4) allele (Corder et al., 1993; Roses et al., 1996). The presence of one or more ε4 alleles confers increased risk for AD development, with one allele increasing the risk of AD development by 47% and two alleles conferring a 90% increase in AD risk (Corder et al., 1993). The presence of an ε4 allele has also been found to increase the rate of transition between the preclinical and symptomatic stages of AD (Vermunt et al., 2019) and the presence of an ε4 allele has been found to be associated with higher amyloid lesion burden (Pletnikova et al., 2018). However, the presence of an ApoE ε4 allele is not sufficient to guarantee AD development and cannot be used as a singular indicator of AD development or preclinical AD.

A potential avenue for detecting risk for cognitive decline prior to the onset of severe neurodegeneration is through olfactory assessment (Murphy, 2019). There is significant evidence that olfactory decline occurs prior to the onset of clinical symptoms and may be a sensitive indicator of preclinical AD pathology. The most studied olfactory assessment is odor identification. Poorer odor identification is associated with AD risk, cognitive decline, and onset of mild cognitive impairment (MCI) (Calhoun‐Haney & Murphy, 2005; Conti et al., 2013; Devanand et al., 2010; Dintica et al., 2019; Graves et al., 1999; Growdon et al., 2015; Hagemeier et al., 2016; Murphy, 2019). Less is known about the utility of other olfactory assessments to detect early olfactory declines in preclinical AD, but there exists some evidence that recognition memory for odors (Dhilla‐Albers et al., 2016; Gilbert & Murphy, 2004; Larsson et al., 2016) and perceived familiarity of odors (Murphy, 2019; Wheeler & Murphy, 2021) may provide additional predictive utility.

In addition to psychophysical evidence, there is significant evidence that the neural processing of odors is impaired in the early stages of the AD disease processes. Studies of olfactory processing in clinically normal populations have found that olfactory processing is related to activity in medial temporal lobe regions (MTL; such as the hippocampus, perirhinal cortex, entorhinal cortex, parahippocampal gyrus, and amygdala), the insular cortex, the orbitofrontal cortex, thalamus, and piriform cortex (Arnold et al., 2020; Lu et al., 2019; Rai et al., 2021; Saive et al., 2014). Studies of the neuropathological staging of AD have found that the earliest presence of neurofibrillary tangles, amyloid plaques, and cellular degeneration appears in the medial temporal lobe, particularly the transentorhinal area and hippocampus, before spreading to neocortical regions in the occipital and parietal lobes (Braak & Braak, 1991). There has been limited research into differences in neural activation levels in those at genetic risk of AD during olfactory processing, but early memory research shows differential activation in olfactory and memory regions including the precuneus, anterior cingulate cortex, middle temporal gyrus, and orbitofrontal cortex, as well as disrupted relationships between activation of MTL and frontal lobe processing regions (Haase et al., 2011; Kapoulea & Murphy, 2020). However, there exists very little research into task‐based functional connectivity (FC) during olfaction in those at risk of AD development.

A promising method for identifying functional networks and understanding neural disruptions in AD is through group spatial independent components analysis (ICA). ICA focuses on identifying functionally connected regions of the brain by separating the signal into spatially independent components and is useful for many applications, including separating noise and signal components (Pruim et al., 2015), identifying large‐scale brain networks, and separating functional networks related to a specific task (Nair et al., 2019). There have been many studies into disruptions to neural components in AD, with many studies finding disrupted resting‐state FC (rsFC) of the default mode network, especially in posterior parietal regions such as the precuneus and inferior parietal lobule (Binnewijzend et al., 2012; Koch et al., 2012; Mondragón et al., 2021). Another study applied machine learning to resting state ICA and found that several independent components (ICs) predicted cognitive status, with medial frontal, sensory–motor, executive control, left dorsal attention, and lateral visual networks best predicting cognitive status (Qureshi et al., 2019).

There has been relatively little research into ICA during olfactory processing (Frasnelli et al., 2012; Karunanayaka et al., 2014; Kollndorfer et al., 2015; Georgiopoulos et al., 2019; Reichert et al., 2017, 2018). In a study with 20 participants, Karunanayaka et al. (2014) found several networks responsive to olfactory processing demands, including networks in the MTL, sensory‐olfactory processing areas, and parietal lobes. These analyses were also followed up with unified structural equation modeling (uSEM), which found that a component in the MTL and a sensory‐olfactory processing component were strongly related. Reichert et al. (2018) found that olfactory processing was related to activity in cerebellar, sensory‐olfactory processing, and occipital networks and that functional recruitment of these networks was related to olfactory deficits (*n* = 48). Geogiopoulos et al. (2019) found that olfactory processing was related to a network involving the piriform cortex, orbitofrontal cortex, thalamus, and insula and that this network was recruited less by participants with Parkinson's disease than by normal controls (*n* = 40).

However, there is a paucity of evidence to determine whether olfactory stimulation prompts differential network FC in those at risk for AD development. In addition, there is not sufficient evidence into whether olfactory FC between and within networks is modulated by task demands. Olfactory deficits observed in the preclinical stages of AD are not understood well at the functional network level, and the degree to which the neural architecture of functional response changes during olfactory processing is relatively unknown. This study sought to elaborate on the existing evidence for olfactory network processing by understanding how the FC of these networks responds to task demands and whether task‐modulated network FC can be used to further elucidate olfactory disruptions observed in those at risk for AD.

## 2 METHODS

### 2.1 Participants

Participants in this study were provided by the UCSD Alzheimer's Disease Research Center (ADRC). During recruitment, participants were excluded from the study if they were found to have any major neurological conditions, a clinical diagnosis of AD or other dementia, MRI contraindications, or any conditions that have a major effect on the sense of smell. Thirty‐nine right‐handed adults completed MRI scanning. Of this sample, 3 were excluded due to high motion and/or noisy imaging data, leaving 36 subjects (20 female, 16 male). All subjects were aged 68 or older (*M* = 76.61, SD = 4.22). All subjects were genotyped for ApoE ε4 allele status, with 16 subjects carrying one ApoE ε4 allele. Two subjects were removed from the final regressions due to missing olfactory testing data. Mean and standard deviations for age, olfactory assessment, cognitive assessment, and years of education for ε4 carriers and non‐carriers can be seen in Table 1. Ethical guidelines for human subjects research were followed. Subjects gave informed consent and the research was approved by Institutional Review Boards at San Diego State University (Protocol number 1633) and University of California San Diego (Protocol number 170289).

**TABLE 1:** Participant demographics and olfactory scores for ε4 carriers and noncarriers.

	Non‐carriers (*N* = 20, 10 females)		Carriers (*N* = 16, 10 females)	
	Mean	SD	Mean	SD
Age	76.8	3.35	76.38	5.21
ADAS‐Cog total score	6.72	3.78	5.02	3.1
Years of education	16.55	2.8	17.5	1.91
SDOIT score	4.25	2.25	4.53	1.64
Mean odor familiarity	4.39	1.69	5.19	2.14

SDOIT, San Diego Odor Identification.

### 2.2 Olfactory assessment

All subjects completed a battery of olfactory and neuropsychological assessments prior to MRI acquisition. During the olfactory assessment, subjects completed a measure of odor identification and odor familiarity. For the odor identification assessment, subjects were administered the San Diego Odor Identification (SDOIT). The SDOIT is a reliable and valid measure of odor identification during which subjects are presented with household odors (for example, peanut butter) and asked to identify the odorant from a set of pictures depicting target and foil odors (Krantz et al., 2009; Murphy et al., 1994). Subjects were also presented with a separate set of 10 household odors which participants rated for familiarity on a scale of 1–10. Mean familiarity was calculated for these odorants.

### 2.3 fMRI acquisition and preprocessing

MR imaging was performed on a GE 3T scanner using an 8‐channel head coil. Functional images were collected using a gradient echo EPI pulse sequence (36 axial slices, field of view = 19.2 cm, resolution 3 × 3 × 3 mm^3^, repetition time = 2 s, echo time = 30 ms, flip angle = 90°). T1‐weighted structural images were also acquired for each subject (field of view = 24 cm, resolution = 0.94 × 0.94 × 1.2 mm^3^, repetition time = 8.2 ms, echo time = 3.18 ms, flip angle = 8°). Data were minimally preprocessed using fMRIPrep v21.0.1 (Esteban et al., 2019) with boundary‐based registration and FreeSurfer surface preprocessing disabled. Following minimal preprocessing, data were smoothed with an 8‐mm Gaussian kernel full‐width half maximum.

### 2.4 Olfactory task design

Subjects were presented with odorants during two runs using an olfactometer. Odorants were presented unirhinally to participants through Teflon tubing that was inserted into the participant's nostril. Task instructions were presented to the participants using a projection screen and a mirror. Participants were instructed to respond using a button box that allowed them to move a cursor up or down on the projected screen. The olfactometer provided a steady stream of compressed air to participants during the entirety of the task to control for any neural processing relating specifically to sensory perception of airflow. During olfactory stimulation events, subjects were administered one of eight possible odorants while being instructed to smell the presented odorant. Odorants were eight concentrated oil formulants designed to simulate common odorants (e.g., coffee). After the stimulus presentation, subjects were presented with four verbal options and asked to move the cursor to select the verbal label corresponding to the odorant being presented. Each olfactory presentation and choice selection event lasted a total of 16 s. Following the presentation of all eight odorants, baseline neural activity was collected during a 16‐s period in which subjects were simply asked to place the cursor in a specific location on the screen to ensure that neural activity relating to the motor movement of the cursor was controlled for. Subjects were presented with all eight odorants four times each per run. Subject responses were coded for hits (correct identifications) and misses (incorrect identifications) using R v4.0.1.

### 2.5 Independent component analyses

Group ICA was conducted on the preprocessed data using the group ICA of fMRI toolbox (GIFT; Calhoun et al., 2001) v3.0 in Matlab version 2021b. The number of components was estimated as 48 using the minimum description length (MDL) criterion and extracted using the infomax algorithm. To ensure the statistical reliability of independent components, ICASSO was used to perform component estimation 10 times. Subject‐specific component spatial maps were back‐reconstructed with the GICA3 algorithm and scaled to z‐scores. To identify task‐relevant components, components were temporally sorted by their correlation with a time‐series of the expected hemodynamic response for each stimulus presentation. Canonical hemodynamic response functions (HRFs) were generated using 3dDeconvolve in AFNI (Ward, 2006) using both a generic subject‐invariant time series of stimulus presentation periods and subject‐specific time series for hits and misses. Pre‐testing of the generic HRF time series indicated that a 10‐s HRF provided the highest mean correlations with task‐relevant components.

Previous studies differ in methodology for retaining task‐relevant components, ranging from selecting an arbitrary number based on correlation/beta values (van der Horn et al., 2015), visual inspections and spectral analysis (Frewen et al., 2017; Griffanti et al., 2017; Jarrahi et al., 2015; Jung et al., 2020; Lee Masson et al., 2020; Xu et al., 2016), or one‐way ANOVAs of beta‐coefficients (Braden et al., 2017; Jarrahi et al., 2015; Xu et al., 2016; Jung et al., 2020). To isolate only components with significant task‐relatedness, we retained components as task‐relevant if the correlation with the generic HRF time‐series was greater than an absolute value of.2. After temporal sorting to determine task relevance, beta‐coefficients specific to hits and misses for each participant were generated using temporal sorting of components using multiple regression with subject‐specific HRF time series.

To assess for task‐modulated FC (TMFC), a hybrid technique of ICA and generalized psychophysiological interaction analysis was implemented. Subject‐specific time series of components that were retained as task‐relevant were extracted for each participant. General linear modeling was then performed separately for each time series. The GLM matrix was designed to include the main effects of hits, misses, and the component time series in addition to interaction terms modeling the multiplicative effect of the component time series and each task condition. The effect of the six rigid‐body motion parameters, their temporal derivatives, quadratic effects of motion parameters and derivatives, and the five aCompCor (Behzadi et al., 2007) components that explained the most variance were included to control for motion and physiological noise sources. Time points at which motion exceeded 1 mm of FD or 2 mm of DVARS were included as spike regressors in the design matrix to control for instances of high motion. High‐pass filtering at 0.01 Hz was also conducted to control for low‐frequency noise sources.

### 2.6 Statistical analysis

To create group‐level spatial maps of all task‐relevant components, each subject's average spatial map across each run was included in a one‐sample *t*‐test. Results were thresholded at a voxel‐level *p*‐value of.0001 and a cluster‐level threshold of 100 voxels.

To examine overall network activation during hits and misses, beta‐coefficients corresponding to HRF response during hits and misses were analyzed for each task‐relevant component using multiple linear regression. SDOIT scores, mean odor familiarity, ApoE ε4 status, biological sex, and age in years were included as predictors. To probe for moderation effects, ApoE x SDOIT, ApoE x odor familiarity, SDOIT x odor familiarity, and SDOIT x odor familiarity x ApoE status interactions were included in the regression. All continuous predictors were mean‐centered and all analyses were conducted in R v4.0.1.

To examine TMFC, subject‐specific spatial maps of the interaction effect between component time courses and hits and misses were evaluated using multiple linear regression. Similar to overall network activation analyses, the main and interaction effects of SDOIT scores, mean odor familiarity, and ApoE ε4 status were examined, controlling for age and sex. All continuous predictors were mean‐centered. The results of these regressions were thresholded at a voxel‐level *p*‐value of.001 and a cluster‐level threshold of 75 voxels. A cluster‐level threshold of 75 was chosen to limit false‐positive rates while still allowing for the detection of significant clusters in the MTL. Given the sample size, the three‐way interaction was considered exploratory and corrections were not applied. Plots of selected interaction effects were created by isolating each subject's TMFC values for significant clusters and plotting estimated regression effects in R v4.0.1.

## 3 RESULTS

### 3.1 Task‐positive components

Five components displayed correlations with an absolute magnitude of greater than.2 and were retained as task‐positive. Component 5 showed the highest correlation with the expected HRF during the task at.33. Components 20 and 25 each showed the next highest correlation values at.27. Finally, components 13 and 29 showed the lowest correlation with the task at.21.

**FIGURE 1:**
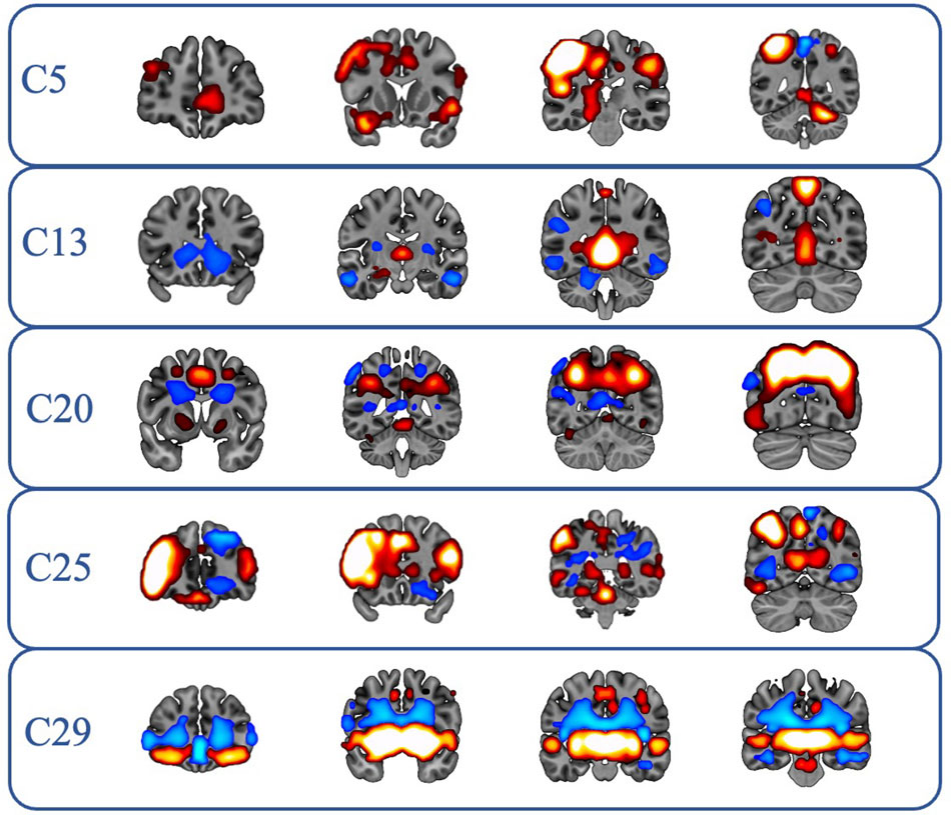
Spatial distributions of task‐positive components. Each row depicts the spatial distribution of an individual component, with rows depicting, in order, components 5, 13, 20, 25, and 29.

The results of the one‐sample *t*‐tests on each component revealed the functional architecture of these networks. Regions of peak connectivity can be seen in Table 2. Spatial distributions of FC for each network can be seen in Figure 1.

**TABLE 2:** Regions significantly associated with each component (voxel‐level *p* = .0001, cluster‐threshold = 100 voxels).

Component	Direction	Regions	Cluster size
5	Positive	Left postcentral gyrus	5555
	Positive	Right cerebellum (VI)	482
	Positive	Left parahippocampal gyrus	221
	Negative	Left precuneus	196
	Positive	Left middle occipital gyrus	169
	Positive	Right middle temporal gyrus	162
	Positive	Right temporal pole	159
	Positive	Left temporal pole	143
	Positive	Right precentral gyrus	129
	Positive	Right mid‐orbital gyrus	127
13	Positive	Cerebellar vermis (4/5)	2462
	Negative	Right caudate nucleus	1043
	Positive	Left precuneus	409
	Negative	Right inferior temporal gyrus	334
	Negative	Left middle temporal gyrus	317
	Negative	Left angular gyrus	234
	Negative	Left superior occipital gyrus	221
	Negative	Right middle frontal gyrus	179
	Negative	Dentate nucleus	137
	Negative	Right cuneus	100
20	Positive	Right cuneus	6033
	Negative	Left middle cingulate cortex	3590
	Negative	Left cuneus	1057
	Positive	Left SMA	870
	Positive	Left insula	471
	Positive	Cerebellar vermis (3)	300
	Positive	Right middle frontal gyrus	205
	Positive	Right middle frontal gyrus	178
	Positive	Right caudate nucleus	148
	Negative	Right inferior frontal gyrus (pars orbitalis)	124
25	Positive	Left inferior frontal gyrus (pars triangularis)	11154
	Negative	Right cuneus	1829
	Positive	Right inferior frontal gyrus (pars triangularis)	1089
	Negative	Right superior frontal gyrus	833
	Positive	Right middle temporal gyrus	707
	Negative	Right middle temporal gyrus	213
	Positive	Right angular gyrus	115
	Positive	Right inferior occipital gyrus	101
29	Positive	Right putamen	6659
	Negative	Right caudate nucleus	6302
	Positive	Right middle cingulate cortex	348
	Negative	Right fusiform gyrus	334
	Positive	Left inferior parietal lobule	203
	Negative	Cerebellar vermis (4/5)	174
	Positive	Right precentral gyrus	161
	Positive	Right fusiform gyrus	158
	Positive	Left fusiform gyrus	131
	Positive	Right inferior frontal gyrus (pars triangularis)	123

### 3.2 Component activation

**FIGURE 2:**
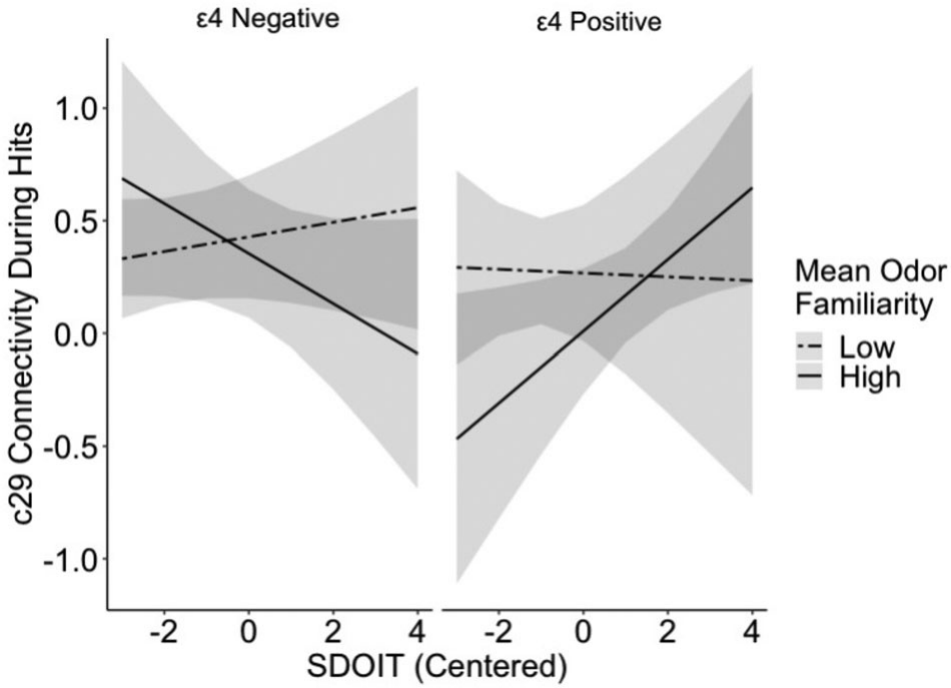
Plot of the marginal mean values for the effect of San Diego Odor Identification (SDOIT) scores activation of component 29 during hits at low and high levels of odor familiarity. ApoE ε4 carriers and non‐carriers are plotted separately. One standard deviation above and below the mean is plotted for each odor familiarity group.

Results of multiple linear regression analyses on the beta‐coefficients for network activations revealed no significant differences for components 5, 13, 20, or 25 during hits and misses. ApoE status was significantly associated with less activation of component 29 during hits (beta = −.25, *p* = .046) but not during misses (beta = −.27, *p* = .077). Results suggest a three‐way interaction between SDOIT scores, ApoE status, and mean odor familiarity during hits (beta = .078, *p*. = .03) and misses (beta = .095, *p* = .03). Plotting of marginal means of this interaction (Figure 2) suggest that ε4 carriers showed greater variability in the relationship between SDOIT scores and network activation as a function of mean odor familiarity relative to ε4 non‐carriers. ε4 carriers with low familiarity ratings showed a general disengagement of component 29 regardless of SDOIT scores, whereas those with intact odor familiarity showed greater network activation with increases in SDOIT scores. ε4 non‐carriers did not show this relationship. ε4 non‐carriers with poorer familiarity showed a slightly positive relationship between SDOIT and network activation, whereas ε4 non‐carriers with high familiarity showed a slightly negative relationship between SDOIT scores and network activation.

### 3.3 Task modulation of component FC

All significant clusters of TMFC related to regressors can be seen in Table S1. There were no significant effects observed on TMFC for components 13 or 25.

**FIGURE 3:**
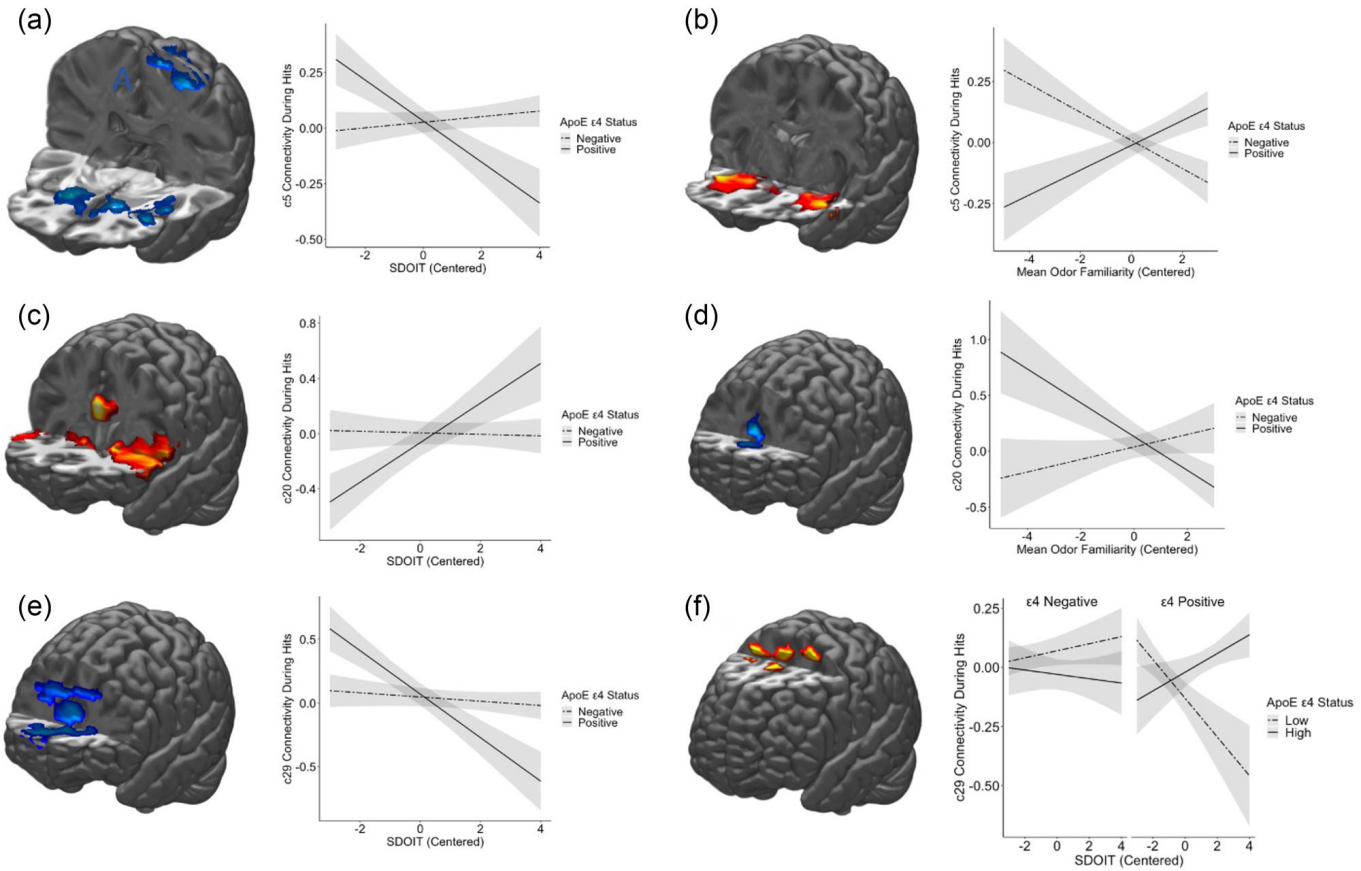
Clusters displaying interaction effects and plotted marginal means. Clusters pictured are the interaction effect of apolipoprotein (ApoE) status and San Diego Odor Identification (SDOIT) scores on component 5 task‐modulated functional connectivity (TMFC) during hits (a), the interaction effect of ApoE status and odor familiarity on component 5 TMFC during hits (b), the interaction effect of ApoE status and SDOIT scores on component 20 TMFC during hits (c), the interaction effect of ApoE status and familiarity on TMFC of component 20 during hits (d), the interaction effect of ApoE status and SDOIT scores on component 29 TMFC during hits (e), and the interaction between ApoE status, SDOIT scores, and odor familiarity ratings on TMFC of component 29 during hits (f).

During hits, component 5 was found to have a negative relationship between reported odor familiarity and TMFC with a cluster in the bilateral orbitofrontal cortex (OFC) and anterior olfactory nucleus (AON; derived from atlas in Echevarria‐Cooper et al., 2022) as well as the right insula, right putamen, and left caudate nucleus. A negative interaction between SDOIT score and ApoE status on TMFC with component 5 during hits was observed for three clusters including the bilateral insula, piriform cortex, AON, and caudate nucleus as well as the left putamen, temporal pole, and inferior frontal gyrus (IFG). Plots of this interaction effect (Figure 3a) suggest that ε4 non‐carriers did not show any relationship between SDOIT status and TMFC with these regions, whereas ε4 carriers showed a negative association, where those with poorer SDOIT scores showed greater TMFC with these regions. A positive interaction between mean odor familiarity and ApoE status on TMFC of component 5 during hits was observed in the right and left hippocampus, IFG, insula, AON, and putamen as well as the left piriform cortex, temporal pole, superior temporal gyrus, the right amygdala, superior orbital gyrus, and caudate nucleus. Plots of the marginal mean TMFC in these clusters (Figure 3b) suggest that ε4 carriers and non‐carriers showed inverse relationships between TMFC and odor familiarity, with ε4 non‐carriers showing a negative relationship, such that those with greater odor familiarity saw lower TMFC values, whereas ε4 carriers showed a positive relationship between TMFC and odor familiarity. During misses, there was a negative interaction effect between ApoE status and SDOIT scores on the TMFC of component 5 observed in the bilateral superior medial gyrus, superior frontal gyrus, middle frontal gyrus, and supplementary motor area.

During hits, there was a significant interaction between SDOIT and ApoE status on TMFC between component 20 and the bilateral caudate, insula, putamen, anterior cingulate cortex (ACC), IFG, and Rolandic operculum. Plots of marginal mean TMFC for this cluster (Figure 3c) revealed that ε4 non‐carriers showed very little to no change in connectivity during hits and that there was no significant relationship between SDOIT score and TMFC, whereas ε4 carriers showed an increase in TMFC between component 20 and this cluster with increasing SDOIT scores. There was a significant interaction effect between odor familiarity and ApoE status on TMFC between component 20 and the bilateral ACC during hits. Plots of marginal mean TMFC for this cluster (Figure 3d) revealed that ε4 non‐carriers showed a negative relationship between TMFC and odor familiarity ratings, whereas ε4 carriers showed a positive relationship between TMFC of this cluster and odor familiarity ratings. No significant effects were observed for component 20 during misses.

During hits, TMFC between component 29 and the bilateral AC, superior medial gyrus, caudate nucleus, and superior frontal gyrus, as well as the left putamen, insula, middle frontal gyrus, and IFG was found to be significantly related to the interaction between ApoE status and SDOIT scores. Plotting the marginal mean TMFC for this cluster (Figure 3e) revealed an inverse relationship to that observed for component 20, where ε4 non‐carriers again showed little to no TMFC change associated with SDOIT score while ε4 carriers showed a negative relationship between SDOIT score and TMFC between component 29 and this cluster. TMFC between component 29 and the bilateral middle frontal gyrus, superior frontal gyrus, and supplementary motor area, as well as the right precentral gyrus, was found to be related to the interaction between SDOIT scores, odor familiarity, and ApoE status during hits. Plotting the marginal mean TMFC for this cluster (Figure 3f) revealed a complex pattern of TMFC across SDOIT scores, familiarity ratings, and ApoE ε4 status. ε4 non‐carriers displayed little change across SDOIT scores or mean odor familiarity ratings. In ε4 carriers, the relationship between frontal lobe connectivity and SDOIT scores was moderated by odor familiarity ratings. ε4 carriers with low familiarity ratings showed a strongly negative relationship between SDOIT ratings and TMFC, whereas those with high familiarity ratings showed a positive relationship between SDOIT scores and TMFC. Those with greater odor familiarity ratings and SDOIT scores showed negative connectivity of this cluster during hits, suggesting some level of inhibitory processing in this region in those with intact identification but poor familiarity. There were no significant clusters of altered TMFC with component 29 during misses.

## 4 DISCUSSION

The components that showed the greatest correlation with the task provide an insight into the greater structure of sensory and cognitive processing networks involved in odor identification. The component with the highest correlation, component 5, was composed of areas related to multisensory integration and sensory–motor processing (Karunanayaka et al., 2015; Starke et al., 2020; Ripp et al., 2018), with the largest cluster of connectivity peaking in the primary sensory cortex. Other areas involved in olfaction and sensory integration, including the parahippocampal gyrus, orbitofrontal cortex, cerebellar lobule VI, AON, and insula (Ferdon & Murphy, 2003; Karunanayaka et al., 2015; Saive et al., 2014) suggest that olfactory processing areas are integrated with other sensory–motor processing areas by a distributed network of nodes in the parietal, occipital, and temporal cortices.

Many regions that comprise component 5 are also significantly related to semantic and verbal processing, with many specifically contributing to odor naming and identification. For example, the left hippocampus is associated with verbal recall (Bonner‐Jackson et al., 2015) and odor identification in AD patients (Murphy et al., 2003). The temporal poles are a hub for semantic information processing (Herlin et al., 2021) and odor naming (Olofsson & Gottfried, 2013; Olofsson et al., 2015). Connections between the temporal poles and hippocampus are associated with subjective memory complaints (Setton et al., 2022). The middle temporal and occipital gyri are also associated with linguistic and semantic processing (Vandenberghe et al., 1996; Whitney et al., 2011; Xu et al., 2016), odor identification (Reichert et al., 2018), and odor‐evoked memories (Arshamian et al., 2013). This suggests that this is a network underlying semantic and linguistic naming processing of odorants in addition to multisensory and sensory–motor integration. This would be consistent with the regions identified in this component as well as with the high correlation with the task. Connections with the sensory and motor cortex may reflect the final integration of this information and motor movements to make a final selection of the odorant's identity.

Components 20 and 25, which displayed slightly lower correlations with the task, may reveal other networks that cooperate to process and identify olfactory information. Component 20 displayed peak connectivity in the right cuneus, supplementary motor area, right middle frontal gyrus, and cerebellum. This component is similar to the occipital processing network observed by Reichert et al. (2018) and may reflect visuospatial and memory processing (Palejwala et al., 2021). Clusters of activation in the insula, thalamus, caudate, AON, precuneus, and orbitofrontal cortex may reflect connectivity between visual, memory, and sensory‐olfactory networks (Meunier et al., 2014; Oh et al., 2018; Palejwala et al., 2021; Saive et al., 2014). This suggests connectivity between visuospatial memory and olfactory processing areas is involved in the odor identification process. This is also supported by peaks in the middle frontal gyrus, a region that is significantly associated with episodic memory retrieval (Rajah et al., 2011).

Component 25 displayed significant FC with areas in the left MTL, insula, orbitofrontal cortex, angular gyrus, and middle temporal gyrus, with peak connectivity being observed in the left IFG. The IFG contributes to olfaction and multisensory integration (Hummel et al., 2010; Olofsson & Gottfried, 2013; Porada et al., 2021). This component also displayed FC with the fusiform gyrus, an area important for odor identification (Masaoka et al., 2021). The middle temporal and angular gyrus being present in this component suggests coordination between sensory integration, semantic processing, and autobiographical episodic recall to identify odorants (Seghier, 2013; Ramanan et al., 2018).

Component 29 included many areas that process olfactory information, including the piriform cortex, insula, orbitofrontal cortex, thalamus, putamen, caudate, AON, and MTL (Saive et al., 2014; Zhou et al., 2019). This network is very similar in structure to sensory‐olfactory networks observed in previous studies (Karunanayaka et al., 2015; Georgiopolous et al., 2019). This component also includes the left inferior parietal lobule, which contributes to processing memory, language, and multisensory information (Igelström & Graziano, 2017; Porada et al., 2021). This component is most likely handling sensory processing of olfactory stimuli in the anterior limbic system, MTL, basal ganglia, OFC, piriform cortex, and insula, regions that have all been previously related to olfactory processing (Fernandez‐Ruiz et al., 2003; Haase et al., 2011; Meunier et al., 2014; Oh et al., 2018; Saive et al., 2014). Previous studies have found odor identification to have a greater cognitive component compared to some other olfactory abilities, namely olfactory threshold (Murphy, 2019). This is supported by component 29 having lower correlations than components 5, 20, and 25. Component 29 processes the sensory‐olfactory information of the odorants being presented, whereas components 5, 20, and 25 integrate this sensory‐olfactory information with cognitive and multisensory information to make decisions about the identity of an odorant.

Component 13 showed an interesting result relative to the other components observed in this study, with most of the peaks indicating areas of negative FC. However, many of the regions negatively associated with this component are areas of peak FC in other components, for example, the right cuneus, right middle frontal gyrus, and caudate. Regions of positive FC included the precuneus, cerebellum, and hippocampus. The areas of positive FC are areas heavily associated with memory and AD. The hippocampus is well known for its role in memory processing (Bonner‐Jackson et al., 2015) and is affected by early AD pathology (Braak & Braak, 1997). Connectivity between the precuneus and hippocampus is altered in AD (Kim et al., 2013). Component 13 may reveal the contributions of posterior hippocampal and precuneus connections to odor identification.

Many of the regions showing peak connectivity during odor identification are similar to regions observed in previous GLM studies of olfaction, including the cerebellum (Ferdon & Murphy, 2003), precuneus (Sunwoo et al., 2015; Chen et al., 2018; Kapoulea & Murphy, 2020), middle temporal and occipital gyri (Arshamian et al., 2013), temporal poles (Olofsson & Gottfried, 2013; Olofsson et al., 2015), OFC (Saive et al., 2014), inferior temporal gyrus (Kjelvik et al., 2012; Suzuki et al., 2001), supplementary motor area (Ruser et al., 2021), insula (Saive et al., 2014), basal ganglia (Fernandez‐Ruiz et al., 2003; Meunier et al., 2014; Oh et al., 2018), IFG (Hummel et al., 2010), inferior parietal lobule (Cerf‐Ducastel & Murphy, 2006), and MTL (Saive et al., 2014).

The regressions of task‐related beta coefficients provide some evidence supporting the use of methods that focus less on overall network activation and instead on the connectivity of specific regions in a component. Non‐significant results observed for overall activation in response to olfactory stimulation for components 5 and 20 despite the presence of alterations in TMFC during hits suggest that disruptions to neural functioning in occipitoparietal processing and multisensory‐semantic integration networks may not reach the level of overall impaired activation of the whole network.

In contrast, there were significant differences observed for activation levels of component 29. Sensory‐olfactory network engagement was lower for ε4 carriers during hits but not misses, showing that ε4 carriers show lesser sensory‐olfactory network engagement even when controlling for olfactory performance. ε4 carriers with poorer perceived familiarity did not show a relationship between sensory‐olfactory network engagement and SDOIT scores, whereas those with intact familiarity showed greater engagement with greater SDOIT scores. These results suggest that ε4 carriers with poorer olfaction show general disengagement of sensory‐olfactory processing networks during odor identification judgments, contrasting with a less extreme and inverted relationship in ε4 non‐carriers. Poorer odor familiarity predicts conversion to aMCI and conversion from aMCI to AD in ε4 carriers, while poorer SDOIT scores and odor familiarity together predict conversion from aMCI to AD in ε4 carriers (Wheeler & Murphy, 2021). Our results suggest that this effect could be related to less engagement of the sensory‐olfactory processing networks in the early stages of olfactory impairment, at least in a sample of subjects without a diagnosis of probable AD. This suggests that disruptions to olfaction that predict risk for aMCI and AD may also predict disruption of the flow of sensory information to cognitive processing networks through sensory‐olfactory network disruptions prior to the onset of dementia.

Differences in TMFC of components 5, 20, and 29 allowed for much greater insight into how the accumulation of genetic risk and olfactory impairment disrupt the connectivity of occipitoparietal, multisensory‐semantic, and sensory‐olfactory networks that contribute to odor identification. For instance, participants reporting greater familiarity with odors showed less TMFC of olfactory processing areas in the OFC, insula, IFG, AON, and basal ganglia with component 5 during hits. ε4 carriers with poorer SDOIT scores showed greater connectivity between component 5 and sensory‐olfactory regions (including the insula, AON, and piriform cortex), the ACC, the precuneus, and the left temporal pole. Many of these regions are implicated as important for AD and odor identification, especially the AON (Saiz‐Sanchez et al., 2020; Ubeda‐Bañon et al., 2020), temporal pole (Herlin et al., 2021; Olofsson & Gottfried, 2013; Olofsson et al., 2015; Setton et al., 2022), and precuneus (Kim et al., 2013). Altered connectivity between multisensory‐semantic processing networks and semantic, sensory, and parietal memory‐processing areas may contribute to increased risk for AD conversion in ε4 carriers with poorer SDOIT scores.

In contrast, lesser connectivity was observed in ε4 carriers with poorer SDOIT scores between the occipitoparietal processing network and olfactory processing areas in the insula, along with basal ganglia and IFG areas important for multisensory processing. This was accompanied by lesser ACC and frontal connectivity. This suggests that, in non‐demented ε4 carriers, poor odor identification may relate to a disruption of reciprocal connectivity between sensory‐olfactory areas and occipitoparietal processing networks. The overlap in differences for interactions between ApoE status and SDOIT scores suggests that the ACC serves as a neural processing hub that unites sensory‐olfactory and occipitoparietal processing networks. The ACC works in combination with the OFC, hippocampus, and prefrontal cortex to aid in memory processing (Rolls et al., 2022) and modulates attention to olfactory stimuli via connections to primary olfactory neurons (García‐Cabezas & Barbas, 2015). This suggests that the ACC has a mediating role in the interface between sensory‐olfactory networks and occipitoparietal processing networks. Poorer odor identification in ε4 carriers may reflect a greater disconnect between these networks.

Differences in TMFC suggest that lower familiarity in ε4 carriers is related to lesser connectivity between multisensory‐semantic integration network hubs and sensory‐olfactory areas, in addition to memory areas in the MTL. This is accompanied by greater connectivity between occipitoparietal networks and the ACC during odor identification. This suggests that a decreased sense of familiarity for odors, in the absence of poorer odor identification, is a warning sign of lesser connectivity between multisensory‐semantic networks, occipitoparietal networks, memory networks, and sensory‐olfactory processing hubs.

Considering the importance of temporal pole and hippocampal connectivity in AD risk, memory, and olfaction (Herlin et al., 2021; Olofsson & Gottfried, 2013; Olofsson et al., 2015; Saive et al., 2014; Setton et al., 2022), this suggests that combining multiple odor performance scores with other risk factors may provide greater evidence of AD risk.

Likewise, lower familiarity may signify a greater reliance on connections between occipitoparietal networks and the ACC to modulate olfactory attention through cognitive control mechanisms. This suggests sensory‐olfactory regions are heavily impacted by the combination of olfactory and genetic risk, which manifests as reduced connectivity between multisensory‐semantic networks and upregulation of olfactory attentional processes in the ACC by occipitoparietal networks. A consequence of this disruption in connectivity could be a reduced involvement of the sensory‐olfactory processing network and the MTL during odor identification.

Finally, the interaction between odor identification, odor familiarity, and ApoE status suggests how the interaction of genetic risk and olfactory function is associated with connections between the frontal cortex and sensory olfactory processing networks. ε4 carriers with poorer familiarity and SDOIT scores showed greater connectivity between the frontal cortex and the sensory‐olfactory processing network. In contrast, ε4 carriers with intact familiarity and poor SDOIT scores showed slightly negative TMFC, whereas ε4 non‐carriers showed very little relationship between connectivity and either familiarity or identification. Connectivity between the orbitofrontal cortex and superior frontal gyrus predicts olfactory impairments in AD, with AD patients showing lower connectivity with worse olfactory impairment (Lee et al., 2020). Our results significantly extend these findings by suggesting that, in non‐demented ε4 carriers with poorer familiarity, poorer SDOIT scores predict greater connectivity between the middle and superior frontal gyri. This suggests that decreased frontal connectivity in clinical AD is preceded by a phase in which greater cognitive control is exerted by the frontal lobe to successfully identify odorants. However, as AD pathology progresses, damage to the MTL and sensory‐olfactory processing regions in the OFC, AON, insula, and thalamus disrupts cognitive control efforts from the frontal lobe, resulting in lesser connectivity.

Although these findings are limited by sample size, they should motivate further investigation of olfactory dysfunction and AD risk factors in nondemented elderly populations.

## 5 CONCLUSIONS

In conclusion, the findings contribute to the clarification of the neurocognitive structure of odor identification processing and disruptions associated with the combination of genetic risk and olfactory impairment. The results suggest that poor odor familiarity and odor identification in ε4 carriers can be an indicator of multi‐network dysfunction. Odor identification involves the coordination of sensory processing, integration, and cognitive networks. The sensory‐olfactory processing network is most at risk, and connections between regions in this network and regions important for memory, semantic processing, visuospatial processing, and multisensory integration are impacted by genetic risk and olfactory impairment. Connectivity with the MTL, ACC, OFC, insula, piriform cortex, and frontal cortex may be most at risk due to their importance in the olfactory processing network. These findings are limited by sample size but should motivate further investigation of olfactory dysfunction and AD risk factors in nondemented elderly populations. Previous research has also shown that biological sex has an effect on AD development (Nebel et al., 2018) and olfaction (Sorokowski et al., 2019). While sex was controlled for in all analyses, this study did not investigate sex as a primary variable of interest. Further research investigating sex may enhance the current understanding of olfactory network connectivity. Regardless, the current study suggests that odor identification and familiarity assessment in ε4 carriers may contribute to the predictive value of risk for MCI and AD due to the breakdown of sensory‐cognitive network integration.

## AUTHOR CONTRIBUTIONS

**Conner Frank**: Conceptualization; investigation; writing—original draft; visualization; formal analysis; methodology. **Abigail Albertazzi**: Investigation; writing—review and editing. **Claire Murphy**: Conceptualization; investigation; funding acquisition; methodology; writing—review and editing; supervision.

## CONFLICT OF INTEREST STATEMENT

The authors declare no conflicts of interest.

### PEER REVIEW

The peer review history for this article is available at https://publons.com/publon/10.1002/brb3.3524

## Supporting information


**Supplementary Table 1**: Clusters of significant differences in task‐modulated functional connectivity (voxel level p >.001, cluster threshold = 75 voxels).

## Data Availability

The data that support the findings of this study are available from the corresponding author upon reasonable request.
