# Supplementary material for: The effect of the apolipoprotein E ε4 allele and olfactory function on odor identification networks
Source: Brain Behav. 2024 May 3;14(5):e3524. doi: 10.1002/brb3.3524 (PMC11069025; doi:10.1002/brb3.3524)
Supplement: Supplementary file 1 — Supplementary Table 1: Clusters of significant differences in task‐modulated functional connectivity (voxel level p >.001, cluster threshold = 75 voxels). [file BRB3-14-e3524-s001.docx]

**Supplementary Table 1**: Clusters of significant differences in task-modulated functional connectivity (voxel level p > .001, cluster threshold = 75 voxels).

| **Component (Condition)** | **Effect** | **Direction** | **Regions** | **Cluster Size** |
| --- | --- | --- | --- | --- |
| **5 (Hits)** | Familarity | Negative | L Olfactory Cortex | 221 |
|  |  |  | L OFC |  |
|  |  |  | R OFC |  |
|  |  |  | R Olfactory Cortex |  |
|  |  |  | R IFG |  |
|  |  |  | R Insula |  |
|  |  |  | R Putamen |  |
|  |  |  | L AON |  |
|  |  |  | R AON |  |
|  |  |  | L Caudate Nucleus |  |
|  | SDOIT * APOE Status | Negative | L Insula | 391 |
|  |  |  | L Piriform |  |
|  |  |  | L Caudate Nuclesu |  |
|  |  |  | L IFG |  |
|  |  |  | L Temporal Pole |  |
|  |  |  | L Putamen |  |
|  |  |  | L Olfactory Cortex |  |
|  |  |  | L AON |  |
|  |  | Negative | R Mid Orbital Gyrus | 257 |
|  |  |  | R Piriform |  |
|  |  |  | R Insula |  |
|  |  |  | R Caudate Nucleus |  |
|  |  |  | R Olfactory Cortex |  |
|  |  |  | R AON |  |
|  |  | Negative | L Postcentral Gyrus | 196 |
|  |  |  | L SPL |  |
|  |  |  | L Precentral Gyrus |  |
|  |  |  | L SPL |  |
|  |  |  | L Paracentral Lobule |  |
|  |  |  | L Precuneus |  |
|  | Familiarity * APOE Status | Positive | R IFG | 781 |
|  |  |  | R Piriform |  |
|  |  |  | R Insula |  |
|  |  |  | R Putamen |  |
|  |  |  | R Caudate Nucleus |  |
|  |  |  | R Olfactory Cortex |  |
|  |  |  | R Superior Orbital Gyrus |  |
|  |  |  | R Amygdala |  |
|  |  |  | L Olfactory Cortex |  |
|  |  |  | L Caudate Nucleus |  |
|  |  |  | R Hippocampus |  |
|  |  |  | L AON |  |
|  |  |  | R AON |  |
|  |  |  | R Pallidum |  |
|  |  | Positive | L Temporal Pole | 451 |
|  |  |  | L Piriform |  |
|  |  |  | L Hippocampus |  |
|  |  |  | L Insula |  |
|  |  |  | L IFG |  |
|  |  |  | L STG |  |
|  |  |  | L Olfactory Cortex |  |
|  |  |  | L Putamen |  |
| **5 (Miss)** | APOE * SDOIT | Negative | R Superior Medial Gyrus | 928 |
|  |  |  | L Middle Frontal Grysu |  |
|  |  |  | L Precentral Gyrus |  |
|  |  |  | R Middle Frontal Gyrus |  |
|  |  |  | L SFG |  |
|  |  |  | R SFG |  |
|  |  |  | L Superior Medial Gyrus |  |
|  |  |  | R SMA |  |
|  |  |  | L SMA |  |
| **20 (Hits)** | SDOIT * APOE | Positive | L Caudate | 696 |
|  |  |  | L IFG |  |
|  |  |  | L Insula |  |
|  |  |  | L Putamen |  |
|  |  |  | L Rolandic Operculum |  |
|  |  |  | L Postcentral Gyrus |  |
|  |  |  | L Caudate Nucleus |  |
|  |  |  | L Precentral Gyrus |  |
|  |  |  | L IFG |  |
|  |  | Positive | R Middle Cingulate Cortex | 381 |
|  |  |  | L ACC |  |
|  |  |  | R ACC |  |
|  |  |  | R Middle Cingulate Cortex |  |
|  |  |  | R Superior Medial Gyrus |  |
|  |  | Positive | R Caudate Nucleus | 213 |
|  |  |  | R Putamen |  |
|  |  |  | R Pallidum |  |
|  |  |  | R Insula |  |
|  |  | Positive | R IFG | 84 |
|  |  |  | R Rolandic Operculum |  |
|  |  |  | R Insula |  |
|  | Familiarity * APOE | Negative | L ACC | 152 |
|  |  |  | R ACC |  |
|  |  |  | L Superior Medial Gyrus |  |
|  |  |  | R Middle Cingulate Cortex |  |
|  |  |  | L Middle Cingulate Cortex |  |
| **29 (Hits)** | APOE * SDOIT | Negative | L ACC | 1220 |
|  |  |  | L Superior Medial Gyrus |  |
|  |  |  | R Superior Medial Gyrus |  |
|  |  |  | R SFG |  |
|  |  |  | R ACC |  |
|  |  |  | L Caudate Nucleus |  |
|  |  |  | L SFG |  |
|  |  |  | R MFG |  |
|  |  |  | R Caudate Nucleus |  |
|  |  |  | R Middle Cingulate Cortex |  |
|  |  |  | L Putamen |  |
|  |  |  | L Insula |  |
|  |  |  | L Middle Frontal Gyrus |  |
|  |  |  | L Rolandic Operculum |  |
|  |  |  | L IFG |  |
|  | 3 Way Interaction | Positive | R MFG | 211 |
|  |  |  | R SMA |  |
|  |  |  | R SFG |  |
|  |  |  | L SFG |  |
|  |  |  | L SMA |  |
|  |  |  | R Precentral |  |
|  |  |  | L MFG |  |
